# POEMS (Polyneuropathy, Organomegaly, Endocrinopathy, Monoclonal Gammopathy, and Skin Changes) Syndrome Masquerading as Chronic Inflammatory Polyradiculoneuropathy: A Case Report

**DOI:** 10.7759/cureus.31481

**Published:** 2022-11-14

**Authors:** Angel Bayas, Thomas Hammond, Marc A Swerdloff

**Affiliations:** 1 Neurology, Florida Atlantic University Charles E. Schmidt College of Medicine, Boca Raton, USA; 2 Neurology, Marcus Neuroscience Institute, Boca Raton Regional Hospital, Boca Raton, USA

**Keywords:** monoclonal gammopathy, pet scans, elevated csf protein, chronic inflammatory demyelinating polyneuropathy, poems syndrome

## Abstract

POEMS (polyneuropathy, organomegaly, endocrinopathy, monoclonal gammopathy, and skin changes) syndrome is a rare disorder that can mimic chronic inflammatory demyelinating polyradiculopathy (CIDP). In this report, we present a case of a man with a new diagnosis of POEMS syndrome and a clinical picture of CIDP. He had prostate cancer (s/p prostatectomy) with known diffuse bony osteosclerotic lesions and a monoclonal gammopathy of undetermined significance (MGUS). The objective of this report is to highlight the importance of recognizing POEMS as a rare condition, differentiating it from CIDP, and initiating treatment as soon as possible. The diagnosis of POEMS can be delayed due to its extensive variety of clinical manifestations, and the extensive workup needed for the diagnosis.

## Introduction

POEMS (polyneuropathy, organomegaly, endocrinopathy, monoclonal gammopathy, and skin changes) syndrome is a rare disorder of unknown cause affecting patients in the fifth or sixth decades of life, with a clinical picture resembling chronic inflammatory demyelinating polyradiculopathy (CIDP). The exact incidence of this condition is unknown. The diagnosis is based on meeting two mandatory, one major, and one minor criteria [1]. The object of this paper is to report the findings of our case, which was a diagnostic challenge due to the preexisting presence of possible metastatic bony sclerotic prostate cancer in our patient, which obscured the presence of sclerotic myeloma. Positron emission tomography (PET) scan technology was used to isolate active neoplasm and direct our attention to proper biopsy sites. Treatment prior to the recognition of the diagnosis was directed toward reversing CIDP. A positive biopsy changed the course of treatment toward eliminating the underlying myeloma. Bone marrow transplant is now used for the treatment of POEMS [2]. However, in our case, this treatment was precluded by the patient's comorbid metastatic cancer.

## Case presentation

**Figure 1:**
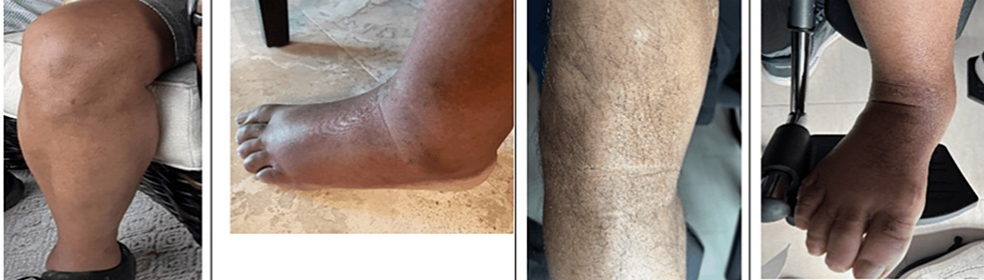
Skin changes: hyperpigmentation, peripheral edema, and hypertrichosis of lower extremities The picture at the far left (1-2-2022) shows the premorbid absence of hypertrichosis. The second picture from the left (2-15-2022) shows dependent edema and the absence of hypertrichosis. Pictures at the far right and second from the right (10-4-2022) show hypertrichosis and hyperpigmentation, respectively. There is less dependent edema due to elevation and support hose

We report a case of POEMS in a 67-year-old, right-handed African American male who presented at the outpatient with the clinical picture of CIDP. He had a history of prostate cancer (s/p prostatectomy) with known diffuse boney osteosclerotic lesions and a monoclonal gammopathy of undetermined significance (MGUS). He also had subacute progression of initially sensory and then motor symptoms with numbness in the balls of his feet, which had spread to the toes, a feeling of walking on cotton, followed by bilateral foot drop, and sensory ataxia. A podiatrist diagnosed arthritis initially. The patient subsequently developed deep vein thrombosis with swelling and was placed on anticoagulant therapy. Over the following months, he presented skin changes, and progressive numbness of the second and third toes bilaterally. The skin changes are depicted in Figure 1.

The patient's condition progressed to his needing a cane to walk to steady himself. He also developed hand numbness. He presented to the clinic with bilateral hip weakness of 4/5 and profound bilateral distal weakness with bilateral foot drop and trace plantar flexion strength. He was areflexic and had 2+ ankle edema. There was stocking decreased appreciation of pain, temperature, and vibratory sensation. He had a high steppage gait. At that time, he progressed to requiring a walker. He was admitted to the hospital with the diagnosis of possible CIDP. Laboratory workup, imagining studies, and spinal fluid studies were performed. CSF showed albumin cytological dissociation, and the protein level in CSF was increased at 104 mg/dL (normal range: 15-60 mg/dL). Cytology was not done.

He was treated with methylprednisolone 20 mg IV once, and IVIG 0.4 g/kg/daily for five days for CIDP. The Hematology Oncology team was consulted to address his prostatic cancer, IgG gammopathy, and sclerotic bone lesions. Laboratory tests (Table 1) and imaging studies were done for the diagnosis of POEMS.

**Table 1:** Laboratory report

Test	Result	Normal range
Free urine lambda light chains	27.98 mg/L (H)	0–3.79 mg/L
Free urine kappa light chains	99.94 mg/L (H)	0–32.9 mg/L
Lambda free light chains, serum	61.39 mg/L (H)	5.7–26.30 mg/L
Kappa free light chains, serum	51.87 mg/L (H)	3.30–19.40 mg/L
Platelets	673,000 per microliter of blood (H)	150,000-450,000 per microliter of blood

**Figure 2:**
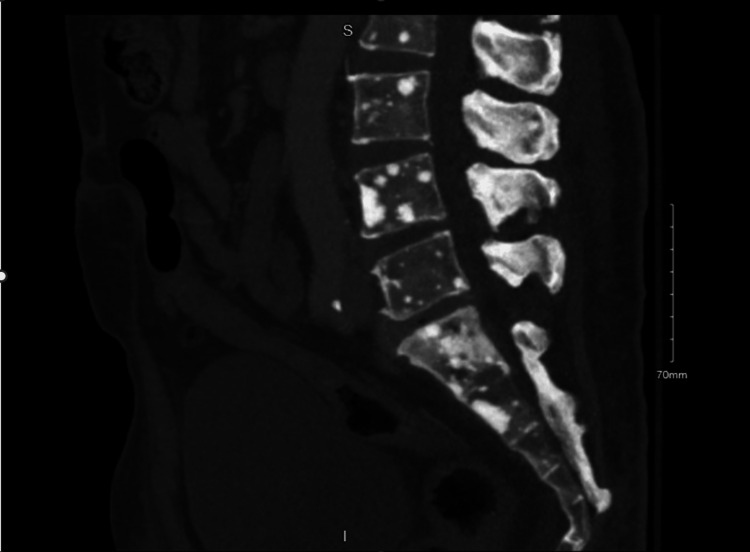
Lateral CT abdomen/pelvis CT: computed tomography

A CT abdomen/pelvis was done, which revealed multiple sclerotic bone lesions scattered throughout the lumbar spine and pelvis suspicious for osteoblastic metastasis (Figure 2).

**Figure 3:**
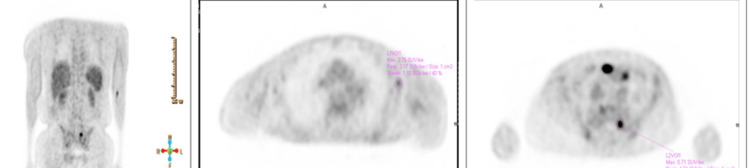
PET scan PET: positron emission tomography

**Figure 4:**
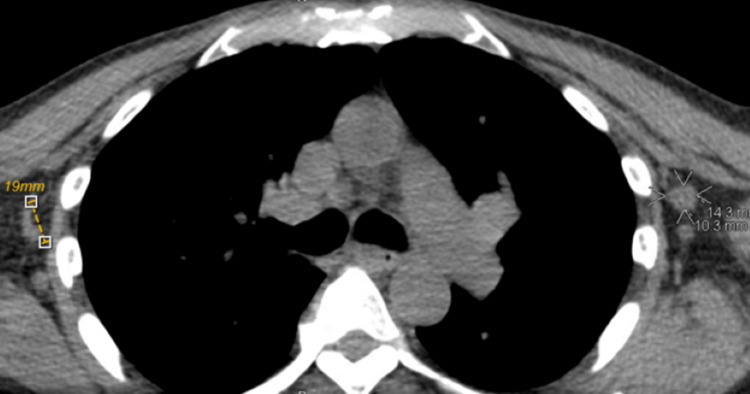
CT chest CT: computed tomography

The patient was discharged following improvement of his numbness but without motor improvement. He was started on higher doses of IVIG. An outpatient PET scan showed diffuse osseous sclerotic lesions, with uptake predominantly within the left, posterior, and medial sacrum representing viable metastatic disease (Figure 3); and a CT chest showed nonspecific uptake involving small bilateral axillary lymph nodes, possibly reactive rather than neoplastic (Figure 4).

**Figure 5:**
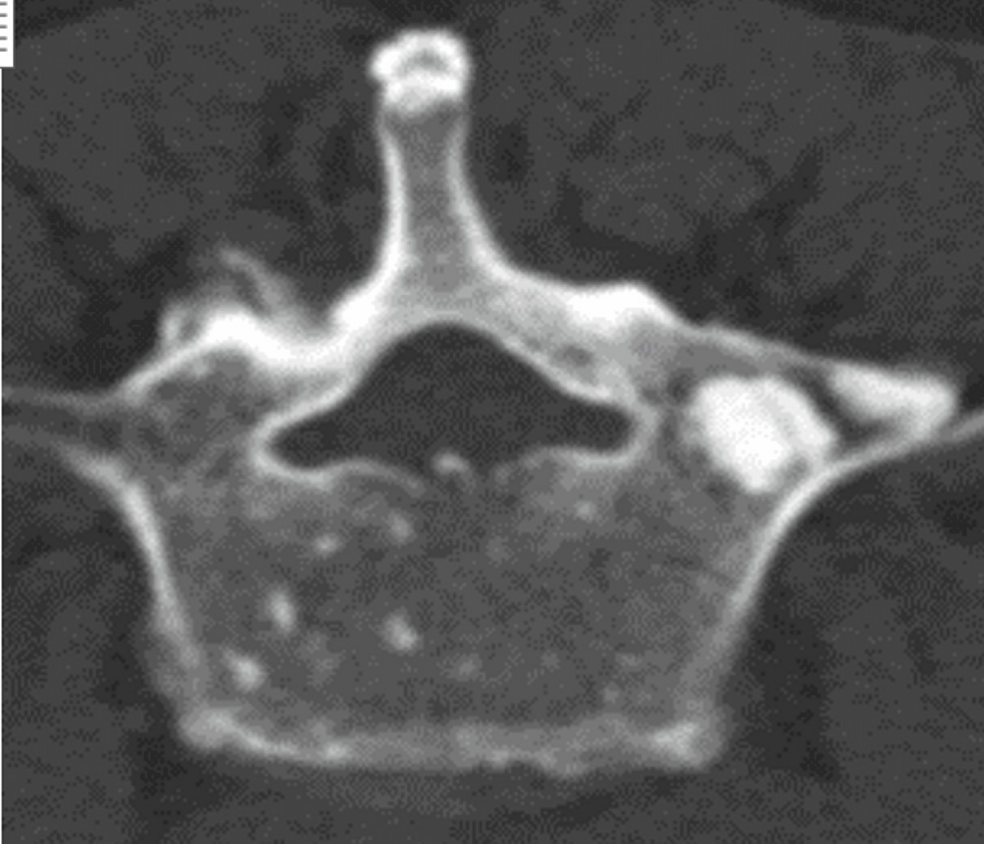
CT-guided bone marrow biopsy of the left iliac bone lesion CT: computed tomography

A CT-guided bone marrow biopsy of the left iliac bone lesion was performed (Figure 5). Pathologic report of the sacral bone lesion was consistent with bone involvement by a plasma cell neoplasm CD138 (+), CD56 (+), and cyclin D1 (-), which were kappa (+) and lambda (+). There was no evidence of prostate carcinoma on two bone marrow biopsies.

**Figure 6:**
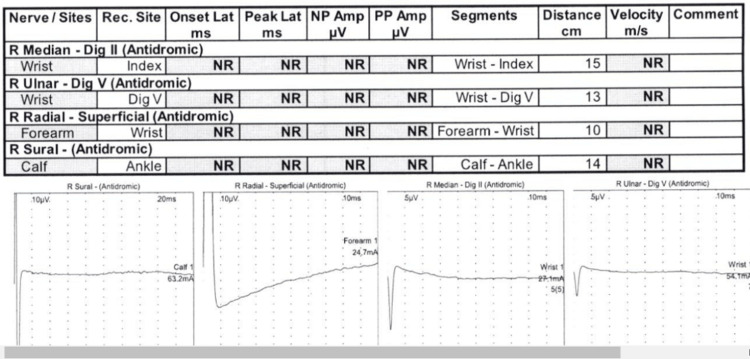
NCS - sensory NCS: nerve conduction study

**Figure 7:**
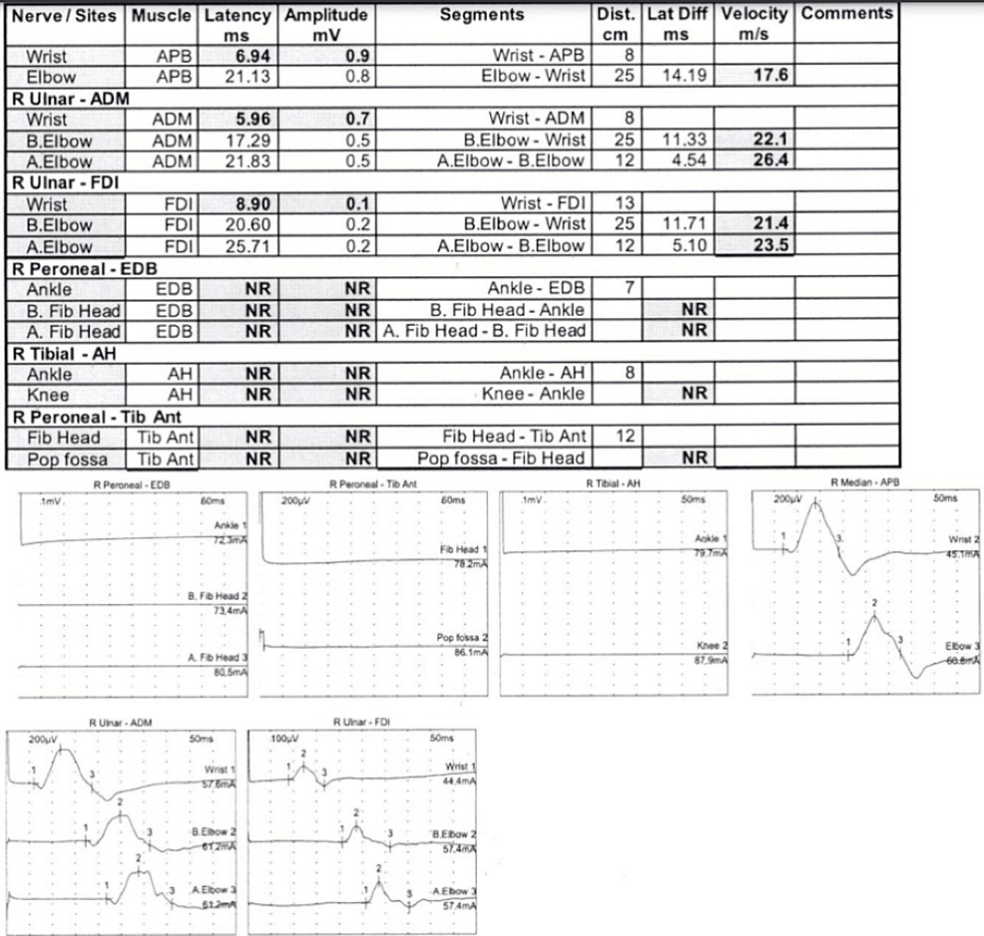
NCS - motor NCS: nerve conduction study

**Figure 8:**
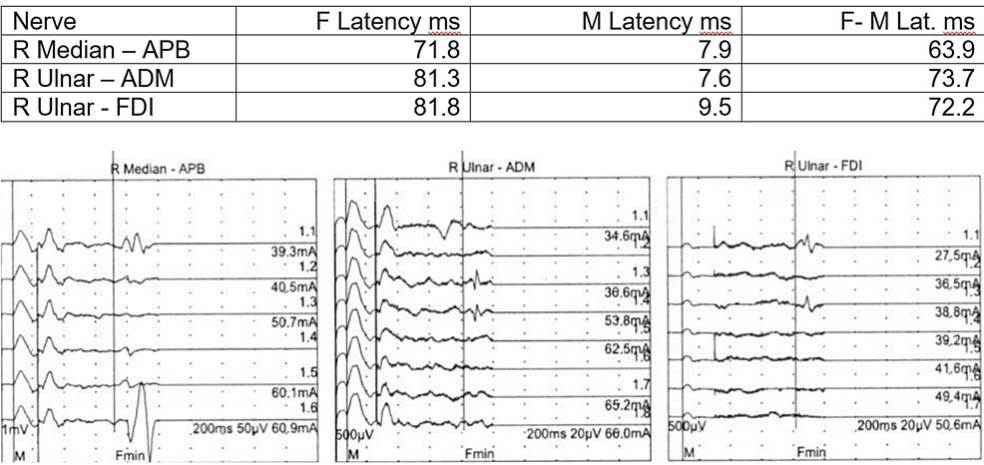
F-Wave

**Figure 9:**
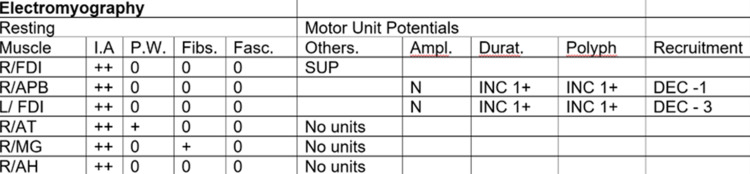
EMG NCS and EMG reports showed absent sensory response from the right median, right ulnar, right radial, and right sural. There was prolonged distal motor latency for the right median and right ulnar. Decreased amplitude of the compound muscle action potential of the right abductor pollicis brevis, right first dorsal interosseous, and right abductor digiti minimi was observed. Markedly decreased motor conduction velocity for the right median and right ulnar nerves was also noted. There was absent motor response from the right peroneal right tibial nerves. These findings are consistent with primarily demyelinating poly radicular neuropathy with only a minimal amount of denervation noted in the right anterior tibialis and the right medial gastrocnemius NCS: nerve conduction study; EMG: electromyography

As an outpatient, he had sensory, motor, and F-wave nerve conduction studies and underwent an EMG (Figures 6-9). There was no need for a nerve biopsy after the completion of the biopsy and electrodiagnostic results.

## Discussion

The diagnosis of POEMS syndrome can be challenging and requires an extensive workup. POEMS syndrome was diagnosed in our patient based on the fulfillment of two mandatory criteria, peripheral neuropathy and monoclonal plasma cell disorder producing majority lambda light chains [3]. The one major criterion is the presence of osteosclerotic bone lesions, and the three minor criteria are thrombocytosis, peripheral edema, and skin changes [1].

Peripheral neuropathy in POEMS syndrome is symmetric and causes tingling, and paresthesia of the distal extremities. These symptoms are progressive and may lead to severe weakness affecting the ability to walk or firmly grip objects [1]. In some cases, patients become wheelchair-dependent, which was the case with our patient. Organomegaly and endocrine abnormalities have also been reported [4], but not found in our case. Osteosclerotic bone lesions can be mixed sclerotic and/or lytic lesions. Some bone lesions are small, solitary, or may present as multiple lesions as in our case (Figure 3). CT and bone scintigraphy are supportive and were done for bone surveys [5]. Since the lesions have variable F-fluorodeoxyglucose (FDG) uptake, a PET scan may not detect all lesions seen on CT [6]. The histopathologic finding of lambda-restricted plasma cell rimming around lymphoid aggregates and megakaryocytic hyperplasia in bone marrow is highly suggestive of POEMS syndrome [7].

Skin changes and volume overload with recurrent, unexplained ascites and peripheral edema are seen in POEMS. Our patient presented hyperpigmentation, hypertrichosis, and peripheral edema, as shown in Figure 1. POEMS can present with thrombocytosis and trigger a hypercoagulative state. Our patient presented deep vein thrombosis of the lower extremity and thrombocytosis with a platelet count of 673.000 per microliter of blood.

Elevated vascular endothelial growth factor (VEGF) levels are an important feature of the POEMS syndrome and have been reported in two-thirds of patients [8]. More than 50% of patients with POEMS have a CSF protein level >100 mg/dL [9]. Our patient had a level of 104 mg/dL. Distal fibrillation potentials are sometimes found on needle EMG in POEMS. However, conduction block is rarely found [10].

There is no standard treatment for POEMS syndrome. Some studies suggest radiation or systemic therapy irrespective of whether the patient has focal or widespread sclerotic bone lesions. A patient with one to three bone lesions and no evidence of bone marrow infiltration may be treated with radiation therapy. Widespread osteosclerotic lesions may be treated with systemic therapy and/or autologous stem cell transplantation (ASCT). However, data on the same is limited [2]. Response to therapy is determined by imaging studies, VEGF levels, and clinical improvement. Polyneuropathy can take up to three to six months to improve, and maximum improvement may take up to three years after treatment [1].

## Conclusions

The diagnosis of POEMS is very challenging and requires an extensive workup and collaboration of multiple specialties. POEMS can be confused easily with CIDP. In our case, the patient’s preexisting prostate cancer complicated the diagnosis of POEMS syndrome. The presence of a paraprotein plus sclerotic boney lesions led us to suspect osteosclerotic myeloma rather than metastatic prostate cancer. We encourage clinicians to report POEMS cases on a regular basis so that guidelines for their treatment can be hopefully established.

## References

[1] S Vincent Rajkumar, MD MD, Robert A Kyle, MD MD (2022). POEMS syndrome. http://www.uptodate.com/contents/poems-syndrome.

[2] Kawajiri-Manako C, Sakaida E, Ohwada C (2018). Efficacy and long-term outcomes of autologous stem cell transplantation in POEMS syndrome: a nationwide survey in Japan. Biol Blood Marrow Transplant.

[3] Abe D, Nakaseko C, Takeuchi M (2008). Restrictive usage of monoclonal immunoglobulin lambda light chain germline in POEMS syndrome. Blood.

[4] Gandhi GY, Basu R, Dispenzieri A, Basu A, Montori VM, Brennan MD (2007). Endocrinopathy in POEMS syndrome: the Mayo Clinic experience. Mayo Clin Proc.

[5] Shibuya K, Misawa S, Horikoshi T (2011). Detection of bone lesions by CT in POEMS syndrome. Intern Med.

[6] Albertí MA, Martinez-Yélamos S, Fernandez A (2010). 18F-FDG PET/CT in the evaluation of POEMS syndrome. Eur J Radiol.

[7] Dao LN, Hanson CA, Dispenzieri A, Morice WG, Kurtin PJ, Hoyer JD (2011). Bone marrow histopathology in POEMS syndrome: a distinctive combination of plasma cell, lymphoid, and myeloid findings in 87 patients. Blood.

[8] D'Souza A, Hayman SR, Buadi F (2011). The utility of plasma vascular endothelial growth factor levels in the diagnosis and follow-up of patients with POEMS syndrome. Blood.

[9] Nozza A (2017). Poems syndrome: an update. Mediterr J Hematol Infect Dis.

[10] Sung JY, Kuwabara S, Ogawara K, Kanai K, Hattori T (2002). Patterns of nerve conduction abnormalities in POEMS syndrome. Muscle Nerve.

